# Impact of race and tumor subtype on second malignancy risk in women with breast cancer

**DOI:** 10.1186/s40064-015-1657-4

**Published:** 2016-01-06

**Authors:** Nicholas Diab, Gary Clark, Lucy Langer, Yunfei Wang, Barbara Hamlington, Laura Brzeskiewicz, Joyce O’Shaughnessy, Sami Diab, Salma K. Jabbour

**Affiliations:** Vanderbilt University, Nashville, TN USA; Array BioPharma, Inc., Boulder, CO USA; Genetic Risk Evaluation and Testing Program, Compass Oncology, Portland, OR USA; Texas Children Hospital, Houston, TX USA; Genetic Risk Evaluation and Testing Program, Rocky Mountain Cancer Centers, Denver, CO USA; Baylor Charles A. Sammons Cancer Center, Texas Oncology, US Oncology, Dallas, TX USA; Rutgers Cancer Institute of New Jersey Rutgers, Rutgers Robert Wood Johnson Medical School, New Brunswick, NJ USA

## Abstract

**Purpose:**

Women with breast cancer are at increased risk of second malignancy (SM). However, the impact of race and the hormone receptor (HR) status of the primary breast tumor on risk of SM are not known. The purpose of this study is to analyze the incidence of SM in women with a history of breast cancer according to race and HR status.

**Methods:**

In the surveillance, epidemiology, and end results database, multiple primary standardized incidence ratio sessions were used to compare the incidence of SM in women with a history breast cancer to the cancer incidence in the general population. Analyses of SM by age, race, and hormone-receptor status were performed using the absolute excess risk (AER) and observed/expected (O/E) ratio.

**Results:**

Younger black women (under the age of 50) were at greater risk of SM with an AER = 76.03 (O/E = 2.3, 95 % CI = 12.19–2.4) compared to younger white women who had an AER = 38.59 (O/E = 1.55, 95 % CI = 1.53–1.58). Older black women (50 years and older) had at an increased risk of SM with an AER = 42.26 (O/E = 1.3, 95 % CI = 1.26–1.34) compared to older white women who had an AER = 11.56 (O/E = 1.07, 95 % CI = 1.06–1.08). Second breast malignancy is the predominant SM in both black and white women. Women with hormone-receptor (HR)-negative breast cancer had higher risk of SMs with an AER = 43.53 (O/E = 1.41, 95 % CI = 1.38– 0.145–3.31) compared to women with HR-positive disease with an AER = 21.43 (O/E = 1.17, 95 % CI = 1.16–0.1.18). In HR-negative women, younger black women had an AER = 96.46 (O/E = 2.99, 95 % CI = 2.70–3.31), younger white women had an AER = 66 (O/E = 2.25, 95 % CI = 2.13–2.36), older black women had an AER = 58.58 (O/E = 1.45, 95 % CI = 1.34–1.57), and older white women had an AER = 20.88 (O/E = 1.14, 95 % CI = 1.11–1.18).

**Conclusions:**

Black breast cancer survivors and women with HR-negative breast cancer are at increased risk of SM, which deserves further evaluation to understand the biological and clinical basis for this increased risk.

## Background

Women with breast cancer are at increased risk of second malignancies (SM) compared to the general population (Berrington de Gonzalez et al. 2010; Schaapveld et al. 2008; Mellemkjaer et al. 2006). As a result of increasing use of adjuvant systemic and local therapies, breast cancer survival rates have improved significantly over the past several decades (5–7). However, prolonged survival times also can carry a burden of increasing SM in surviving patients.

Breast cancer is a heterogeneous disease with varying underlying genetic predispositions and environmental factors resulting in different biological subtypes. The same factors leading to this biological heterogeneity might also lead to differing risks of SM. In addition, racial differences in breast cancer tumor biology and genomics as elegantly reviewed by Daly and Olopade (Daly and Olopade 2015), can lead to differences in the risk of SM. Compared to white (W) women, black (B) women with breast cancer are more likely to be younger at diagnosis (Clarke et al. 2003), carry hormone receptor (HR)-negative breast cancer (Iqbal et al. 2015), present with more advanced stage at diagnosis (Kurian et al. 2010), and have higher rates of BRCA1/2 mutations or polymorphisms (John et al. 2007; Nanda et al. 2005). These clinical and biological differences might also impact the risk of SM. After a first primary breast cancer, no large population-based study has investigated risk of SM by primary breast cancer subtype or race (Molina-Montes et al. 2015).

Based on the differences in the biology and outcomes among the races with breast cancer, and the heterogeneity of breast cancer in general, we hypothesize that the risks of SM are different based on race and the biological subtype of breast cancer. Specifically, we characterized the risk of SMs in patients with a first diagnosis of breast cancer in the surveillance, epidemiology, and end results (SEER) database. We evaluated the risk based on age, race, and HR status.

## Methods

### Study population

The results reported in this study are based on the following SEER database: Incidence—SEER 9 Regs Research Data, Nov 2014 Sub (1973–2012) <Katrina/Rita Population Adjustment >—linked to county attributes—Total U.S., 1969–2013 Counties, National Cancer Institute, DCCPS, Surveillance Research Program, Surveillance Systems Branch, released April 2015, based on the November 2014 submission. This database covers approximately 9.4 % of the US population (based on 2010 census) and the geographic areas and years covered are: San Francisco-Oakland SMSA—1973+; Connecticut—1973+; Detroit (Metropolitan)—1973+; Hawaii—1973+; Iowa—1973+; New Mexico—1973+; Seattle (Puget Sound)—1974+; Utah—1973+; and Atlanta (Metropolitan)—1975+.

Multiple primary standardized incidence ratios (MP-SIR) sessions were generated to compare incidence of SM in the SEER cohort of patients previously diagnosed with breast cancer to the incidence of cancer in the general population. The general population was matched by race and age.

The first primary diagnosis of breast cancer was used and patients were excluded if the diagnosis was obtained solely based on death certificate or autopsy report or if there was no microscopic confirmation of diagnosis. Patients with carcinoma in situ were also excluded. Patients were observed from the diagnosis of breast cancer until the diagnosis of another cancer or death. For the analyses based on hormone receptors (HR), women for whom the summary results of estrogen and/or progesterone receptors were coded as positive or negative in the database were selected. Women whose breast cancer was coded as ER and PR borderline, undetermined, or unknown were excluded. Women with ER-positive or PR-positive cancer were analyzed together as HR-positive disease. For the race analyses, the SEER variables white and black were selected, and other or unknown races were excluded. The terms younger (Y) women and older (O) women in this manuscript refer to women under age 50 and 50 years or older, respectively. The risk of SM are reported for younger white (YW), younger black (YB), older white (OW), and older black (OB). Due to the limited data on HER2 status in the SEER database, the impact of this variable on the risk of SM was not examined.

### Statistical analyses

SEER* Stat 8.2.1, released on 4/08/2015, was used to analyze the observed to expected (O/E) risk of secondary cancers in patients with a first breast cancer diagnosis. The observed incidence is the actual count of events experienced for this subset of patients. The expected incidence is the number of events expected for this subset of general population of women matched by race and age. The O/E risk is the observed count divided by the expected count. The absolute excess risk (AER) is the excess cancer (beyond the expected amount) per 10,000 persons per year. The formula for excess risk is: (Observed count—Expected count) × 10,000/person years at risk. The mean age at exposure is the mean age, in years, at diagnosis of the first cancer among individuals in this subset of the cohort, and the mean age at event is the mean age, in years, at the time of experiencing the SM. 95 % confidence intervals (CI) are reported and the P value is <0.05 if the CIs do not overlap 1.

## Results

### Risk of SM by age

Women with a first diagnosis of breast cancer have a significant increased risk of SM at all sites with higher risk in younger women.

Younger women (n = 126,689, mean age 42.78 years) had 16,492 new SM (AER = 43.06, O/E = 1.64, 95 % CI = 1.62–1.67), and the mean age at time of SM was 54.6 years. In contrast, older women (n = 387,790, mean age 67.2 years) had 58,378 SM (AER = 15.01, O/E = 1.10, 95 % CI = 1.09–1.11), and the mean age at time of SM was 74.4 years.

Younger women had 8696 new second breast malignancy (SBM) (AER = 33.49, O/E = 2.37 95 % CI = 2.32–2.42), and the mean age of SBM was 52.4 while older women had 19.175 SBM (AER = 14.45, O/E = 1.36 95 % CI = 1.34–1.37) and the mean age of SBC was 72.8.

For younger women, the AER was significant for solid tumors (41.91), SBM (33.49), ovarian (2.39), respiratory system (2.29), leukemia (1.11), uterine (1.05), skin (0.61), endocrine cancers (0.57), urinary systems (0.4), soft tissue (0.39), pancreas (0.28), and bone/joint (0.08). For OW, the AER was significant for solid tumors (17.32), SBM (14.45), gynecologic (3.17), skin (0.48), endocrine cancers (0.51), urinary systems (0.4), soft tissue (0.27), and bone/joint (0.08).

### Risk of SM by race

BW women with a first diagnosis of breast cancer had an increased risk of SM compared with WW women regardless of age. YB women (n = 14,888, mean age 41.82 years) had 1897 new SM (AER = 76.03, O/E = 2.3, 95 % CI = 12.19–2.4) and the mean age at time of SM was 52.0 years; YW women (n = 99,946, mean age 42.95 years) had 13,390 new SM (AER = 38.59, O/E = 1.55, 95 % CI = 1.53, 1.58) and the mean age at time of SM was 55.1 years. OB women (n = 29,125, mean age 71.99 years) had 3818 new SM (AER = 42.26, O/E = 1.3, 95 % CI = 1.26–1.34) and the mean age at time of SM was 52.0 years; OW women (n = 334,400, mean age 67.5) had 51,548 new SCs (AER = 11.56, O/E = 1.07, CI = 1.06–1.08) and the mean age at time of SCs was 74.7 years (Table 1).

**Table 1:** Risk of second cancer by age

Under 50					50 and older			
	Observed	O/E	Excess risk	Mean age at event	Observed	O/E	Excess risk	Mean age at event
All sites	16,492	1.64^#^	43.06	54.64	58,378	1.10^#^	15.01	74.42
All solid tumors	15,479	1.68^#^	41.91	54.48	52,829	1.13^#^	17.32	74.2
Oral cavity and pharynx	204	1.25^#^	0.27	55.68	915	1.04	0.11	74.1
Digestive system	1481	1.07^#^	0.61	59.52	11,942	0.98	−0.6	77.01
Colon, rectum and anus	808	0.94	−0.36	59.13	7569	0.98	−0.34	77.15
Pancreas	237	1.22^#^	0.28	60.83	1734	0.95^#^	−0.28	77.03
Peritoneum, omentum and mesentery	45	2.04^#^	0.15	59.65	110	0.94	−0.02	74.85
Respiratory system	1555	1.28^#^	2.29	59.76	7790	0.97^#^	−0.78	74.11
Bones and joints	25	2.01^#^	0.08	53.86	50	1.1	0.01	72.67
Soft tissue including heart	110	2.14^#^	0.39	54.64	334	1.39^#^	0.27	74.43
Skin excluding basal and squamous	525	1.21^#^	0.61	54.16	1604	1.12^#^	0.48	74.49
Breast	8696	2.37^#^	33.49	52.44	19,175	1.36^#^	14.45	72.76
Female genital system	1877	1.35^#^	3.21	54.84	6909	1.19^#^	3.17	73.36
Corpus and uterus, NOS	903	1.21^#^	1.05	56.38	4262	1.36^#^	3.26	73.25
Ovary	720	1.99^#^	2.39	53.05	1785	1.07^#^	0.32	72.97
Urinary system	469	1.14^#^	0.4	59.24	2997	1.04	0.29	75.86
Brain and other nervous system	120	1.06	0.04	54.58	395	0.75^#^	−0.37	72.7
Endocrine system	429	1.25^#^	0.57	52.14	806	1.28^#^	0.51	69.21
Lymphoma	361	0.94	−0.16	58.64	2145	0.91^#^	−0.63	76.54
Leukemia	352	1.90^#^	1.11	53.62	1551	1.08^#^	0.31	75.51

^# ^Indicates P value <0.05

Second breast malignancy was a primary cause of increased risk of SC in both BW women and WW women. The AER for SBM in YB women and YW women was 61.0 and 30.2, respectively, and 31.3 and 12.7 for OB women and OW women, respectively.

When the analyses were limited to non-breast SM (NBSM), YB women had 743 new NBSM (AER = 15.05, O/E = 1.4, 95 % CI = 1.3–1.5) and the mean age at time of SM was 55.2; YW women had 6508 NBSM (AER = 8.43, O/E = 1.19, 95 % CI = 1.16–1.22) and the mean age at time of SCs was 57.38. OB women had 2439 NBSC (AER = 10.95, O/E = 1.10, 95 % CI = 1.06–1.15) and the mean age at time of SMs was 73.20; OW women had 34,819 NBSM (AER = −1.1, O/E = 0.99, CI = 0.98–1.0) and the mean age at time of SCs was 75.4.

### Risk of second malignancy by hormone-receptor status

Women with HR-negative breast cancer had higher risk of SM. Women with HR-negative disease (N = 65,593) had 7442 new SM (AER = 43.53, O/E = 1.41, 95 % CI = 1.38– 0.145–3.31) while women with HR-positive disease (N = 9239,655) had 27,328 new SM (AER = 21.43, O/E = 1.17, 95 % CI = 1.16–0.1.18) (Tables 2, 3, 4, 5, 6).

**Table 2:** Second cancers by race

	Under 50				50 and older			
	White		Black		White		Black	
	O/E	Excess risk	O/E	Excess risk	O/E	Excess risk	O/E	Excess risk
All sites	1.55^#^	38.59	2.30^#^	76.03	1.07^#^	11.56	1.30^#^	42.26
All solid tumors	1.59^#^	37.78	2.37^#^	72.87	1.10^#^	14.23	1.34^#^	42.99
Oral cavity and pharynx	1.25^#^	0.28	0.98	−0.02	1.05	0.12	0.93	−0.13
Digestive system	1.04	0.35	1.08	0.87	0.97^#^	−1.18	1.10^#^	3.71
Colon, rectum and anus	0.91^#^	−0.53	0.98	−0.12	0.97^#^	−0.62	1.07	1.51
Pancreas	1.17^#^	0.23	1.04	0.06	0.92^#^	−0.39	1.07	0.47
Peritoneum, omentum and mesentery	2.11^#^	0.18	1.96	0.07	0.92	−0.03	1.06	0.01
Respiratory system	1.23^#^	1.93	1.69^#^	5.53	0.95^#^	−1.1	1.08	1.73
Bones and joints	1.93^#^	0.08	2.04	0.07	1.1	0.01	1.22	0.03
Soft tissue including heart	2.16^#^	0.4	1.81	0.32	1.34^#^	0.24	1.75^#^	0.54
Skin excluding basal and squamous	1.20^#^	0.67	2.03	0.29	1.11^#^	0.51	1.71^#^	0.38
Breast	2.18^#^	30.16	3.91^#^	60.98	1.30^#^	12.67	1.89^#^	31.31
Female genital system	1.32^#^	3.14	1.47^#^	3.36	1.17^#^	2.93	1.22^#^	3.41
Corpus and uterus, NOS	1.16^#^	0.84	1.70^#^	2.24	1.32^#^	3.02	1.48^#^	3.69
Ovary	1.97^#^	2.5	2.00^#^	1.49	1.07^#^	0.33	0.97	-0.12
Urinary system	1.13^#^	0.38	1.14	0.32	1.02	0.19	1.23^#^	1.68
Brain and other nervous system	1.12	0.1	0.17^#^	−0.34	0.75^#^	-0.4	0.75	−0.23
Endocrine system	1.15^#^	0.35	2.00^#^	1.53	1.25^#^	0.45	1.50^#^	0.82
Lymphoma	0.93	-0.19	0.96	-0.07	0.89^#^	-0.77	1.04	0.16
Leukemia	1.63^#^	0.84	3.95^#^	2.65	1.05	0.23	1.19	0.58

^#^ Indicates P value <0.05

**Table 3:** Second cancers in white and black women under 50 with hormone receptor-negative breast cancer

	White			Black		
	O/E	Excess risk	Mean age at event	O/E	Excess risk	Mean age at event
All sites	2.25^#^	66	50.07	2.99^#^	96.46	48.24
All solid tumors	2.29^#^	63.39	50.01	3.08^#^	91.44	48.18
Oral cavity and pharynx	2.35^#^	1.06	51.39	1.91	0.75	54.79
Digestive system	1.09	0.54	52.48	0.87	−1.07	49.84
Colon, rectum and anus	0.81	−0.68	51.86	0.85	−0.77	49.24
Pancreas	0.85	−0.12	47.3	0.37	−0.66	46.42
Peritoneum, omentum and mesentery	8.12^#^	0.77	53.12	14.86#	0.73	53.09
Respiratory system	1.43^#^	1.9	52.75	1.99^#^	5.44	50.75
Bones and joints	2.71	0.15	55.17	0	−0.07	
Soft tissue including heart	4.07^#^	0.91	52.07	1.07	0.03	32.57
Skin excluding basal and squamous	1.21	0.73	50.86	1.47	0.12	61.09
Breast	3.31^#^	48.31	49.09	5.16^#^	78.93	47.01
Female genital system	2.16^#^	8.9	51.33	2.21^#^	7.06	53.27
Corpus and uterus, NOS	1.39^#^	1.59	54.44	2.06^#^	2.82	57.54
Ovary	4.60^#^	6.89	49.84	3.85^#^	3.48	47.03
Urinary system	1.4	0.78	51.42	0.45	−0.95	54.83
Brain and other nervous system	1.09	0.06	51.41	1.14	0.05	58.33
Endocrine system	1.04	0.12	47.93	1.61	1.04	44.75
Lymphoma	1.07	0.14	53.84	1.09	0.16	53.3
Leukemia	3.00^#^	1.82	47.71	8.48^#^	5.18	47.61

^# ^Indicates P value <0.05

**Table 4:** Second cancers in white and black women 50 years and older with hormone receptor-negative breast cancer

	White			Black		
	O/E	Excess risk	Mean age at event	O/E	excess risk	Mean age at event
All sites	1.14^#^	20.88	72.06	1.45^#^	58.58	68.42
All solid tumors	1.17^#^	22.32	71.85	1.49^#^	57.08	68.31
Oral cavity and pharynx	1.05	0.1	70.44	0.75	−0.42	70.85
Digestive system	1	0.09	75.33	1.26^#^	8.2	70.81
Colon, rectum and anus	0.96	−0.76	75.73	1.14	2.55	70.63
Pancreas	0.98	−0.11	76.25	1.47	2.67	68.67
Peritoneum, omentum and mesentery	1.16	0.07	69.36	0	−0.22	
Respiratory system	1.18^#^	4.15	72.59	1.25^#^	5.51	68.75
Bones and joints	0.75	−0.03	67.09	2.79	0.21	71.17
Soft tissue including heart	1.37	0.25	71.3	1.37	0.26	67.22
Skin excluding basal and squamous	1.06	0.33	71.19	1.9	0.45	68.58
Breast	1.37^#^	15.12	70.74	2.04^#^	36.73	67.02
Female genital system	1.18^#^	2.91	70.41	1.25	3.56	67.77
Corpus and uterus, NOS	1.23^#^	2.07	70.72	1.67^#^	5.39	67.91
Ovary	1.30^#^	1.37	69.62	0.7	−0.96	60.84
Urinary system	0.95	−0.44	72.29	1.23	1.58	68.57
Brain and other nervous system	0.78	−0.33	69.23	1.51	0.43	72.63
Endocrine system	1.19	0.47	65.7	1.4	0.82	61.22
Lymphoma	0.88	−0.8	74.86	1.31	1.21	65.59
Leukemia	1.2	0.77	71.68	1.35	0.92	72.13

^#^ Indicates P value <0.05

In women with HR-negative disease, YB women (N = 3578) had 370 new SM (AER = 96.46, O/E = 2.99, 95 % CI = 2.70–3.31), YW women (N = 14,087) had 1485 SM (AER = 66.00, O/E = 2.25, 95 % CI = 2.13–2.36), OB women (N = 3578) had 590 new SM (AER = 58.58, O/E = 1.45, 95 % CI = 1.34–1.57), and OW women (N = 30,117) had 3520 SM (AER = 20.88, O/E = 1.14, 95 % CI = 1.11–1.18).

In women with HR-positive disease, YB women (N = 5782) had 476 new SC (AER = 63.89, O/E = 2.29, 95 % CI = 2.09–2.5), YW women (N = 3829) had 1485 SC (AER = 36.87, O/E = 1.64, 95 % CI = 1.59–1.7), OB women (N = 12,689) had 1380 new SM (AER = 35.74, O/E = 1.25, 95 % CI = 1.19–1.32), and OW women (N = 161,244) had 20,547 SC (AER = 12.00, O/E = 1.08, 95 % CI = 1.06–1.09).

**Table 5:** Second cancers in white and black women under 50 with hormone receptor-positive breast cancer

	White			Black		
	O/E	Excess risk	Mean age at event	O/E	Excess risk	Mean age at event
All sites	1.64^#^	36.87	52.03	2.29^#^	63.89	50.76
All solid tumors	1.67^#^	35.63	52.08	2.34^#^	60.24	50.54
Oral cavity and pharynx	1.57^#^	0.49	53.33	0.29	-0.59	63
Digestive system	1.05	0.32	54.15	1.25	2.19	51.39
Colon, rectum and anus	0.9	−0.42	53.03	1.05	0.24	50.74
Pancreas	1.41^#^	0.35	56.16	1.33	0.35	56
Peritoneum, omentum and mesentery	1.42	0.05	56.73	0	−0.05	
Respiratory system	1.37^#^	1.83	55.38	1.56^#^	3.15	52.2
Bones and joints	1.62	0.06	47.4	3.69	0.17	49.25
Soft tissue including heart	1.96^#^	0.3	51.14	1.91	0.34	51.53
Skin excluding basal and squamous	1.42^#^	1.48	50.76	3.58	0.69	53.21
Breast	2.22^#^	27.6	51.43	3.53^#^	49.01	49.75
Female genital system	1.24^#^	1.97	52.87	1.32	1.91	55.19
Corpus and uterus, NOS	1.39^#^	1.78	54.02	1.84^#^	2.28	56.48
Ovary	1.23^#^	0.48	51.05	0.96	−0.06	49.73
Urinary system	1.35^#^	0.75	54.28	1.88^#^	1.56	51.6
Brain and other nervous system	1.40^#^	0.28	51.19	0	−0.35	
Endocrine system	1.26^#^	0.78	49.27	2.58^#^	2.77	47.17
Lymphoma	1.04	0.09	53.13	1.04	0.08	54.76
Myeloma	0.94	−0.03	56.76	1.14	0.15	57.99
Leukemia	1.89^#^	0.88	47.48	3.04^#^	1.44	52.46

^#^ Indicates P value <0.05

**Table 6:** Second cancers in white and black women 50 years and older with hormone receptor-positive breast cancer

	White			Black		
	O/E	Excess risk	Mean age at event	O/E	Excess risk	Mean age at event
All sites	1.08^#^	12	74.31	1.25^#^	35.74	72.08
All solid tumors	1.10^#^	13.55	74.12	1.29^#^	36.26	71.94
Oral cavity and pharynx	1.07	0.16	74.7	1.24	0.43	72.9
Digestive system	0.99	−0.38	76.81	1.01	0.39	74.51
Colon, rectum and anus	1	0.09	77	0.97	−0.51	75.17
Pancreas	0.98	−0.12	76.64	1.05	0.33	73.4
Peritoneum, omentum and mesentery	1.02	0.01	75.19	1.15	0.03	63.92
Respiratory system	0.99	−0.37	74.44	1.02	0.48	72.78
Bones and joints	1.05	0.01	73	2.02	0.13	69
Soft tissue including heart	1.29^#^	0.22	74.04	1.92	0.68	68.95
Skin excluding basal and squamous	1.11^#^	0.61	73.4	2.54^#^	0.86	77.64
Breast	1.21^#^	8.91	72.86	1.74^#^	27.13	69.99
Female genital system	1.22^#^	3.64	73.5	1.27^#^	4.04	71.31
Corpus and uterus, NOS	1.43^#^	3.91	73.17	1.49^#^	4.03	71.26
Ovary	1.01	0.05	73.69	1.11	0.37	70.41
Urinary system	1.07^#^	0.62	74.99	1.23	1.74	74.27
Brain and other nervous system	0.80^#^	−0.32	73.04	0.56	-0.4	77.34
Endocrine system	1.34^#^	0.77	68.5	1.69^#^	1.37	69.28
Lymphoma	0.89^#^	−0.8	75.96	0.91	−0.37	70.93
Leukemia	1.15^#^	0.62	75.06	1.39	1.19	71.48

^# ^Indicates P value <0.05

### Risk of second breast malignancy in women with first non-breast malignancy

The risk of breast cancer as a second event (BCSE) after a first diagnosis of non-breast cancer was evaluated. Having reciprocal increased risk supports the presence of shared etiology for both cancers. Black women (n = 92,160) had 1508 new breast cancers as a second event (AER = 3, O/E = 1.11, 95 % CI = 1.06–1.17) while the risk was not elevated in white women when all sites of first cancers are grouped together.

In black women, the risk of SBM was elevated in women with non-basal/non-squamous skin cancer (n = 40, AER = 20.31, O/E = 1.93), ovarian cancer (n = 69, AER = 8.47, O/E = 1.36), thyroid cancer (n = 87, AER = 6.62, O/E = 1.3), and lymphoma (n = 110, AER = 8.66, O/E = 1.39).

In white women, the risk of SBM was elevated in women with soft tissue cancer (n = 196, AER = 4.11, O/E = 1.16), non-basal/non-squamous skin cancer (n = 2015, AER = 1.64, O/E = 1.06), thyroid (n = 1245 = , AER = 3.0, O/E = 1.13), and lymphoma (n = 1553, AER = 3.45, O/E, 1.12), but not ovarian cancer.

## Discussion

To our knowledge, this is the first detailed report demonstrating an increased risk of SM in patients with primary breast cancer based on race, and HR status of breast cancer. The risk of SM was higher in black women than white women for both SBM and NBSM. This higher risk of SM in black women and HR-negative women might be related to underlying genetic variation in DNA repair. In a population-based, multi-ethnic series of female breast cancer patients <65 years at diagnosis who were enrolled at the Northern California site of the Breast Cancer Family Registry, the BRCA1 mutation frequency was 16.7 % in black women diagnosed under age 35 versus 7.2 % in non-Hispanic white patients without Ashkenazi Jewish ancestry. Women with triple-negative breast cancer have higher frequency of BRCA1/2 mutation as well. The potential for higher frequency of impaired DNA repair mechanisms in black women and in HR-negative women (Bartkova et al. 2008) might lead to poor DNA repair after exposure to radiation and systemic therapy and an increased risk of SM. In addition, it is known that higher body mass index is associated with higher incidence of cancer (Renehan et al. 2008; Bhaskaran et al. 2014). BW with breast cancer have been shown to have statistically more comorbidities and higher average body mass index (Tammemagi et al. 2005). Therefore, weight and other comorbidities, which were not controlled-for in this analysis, may play a role in the statistically increased risk of SM.

The strength of the SEER database for statistical analysis is the long-term follow-up of large numbers of women with breast cancer, the availability of data on hormone receptor status and race, and the high quality of the database itself. The SEER Program is viewed as the standard for quality among cancer registries around the world. Each SEER program registry must meet specifically defined data quality goals on an ongoing basis (http://seer.cancer.gov).

However, the SEER registry is limited in that it does not report data on the use of adjuvant systemic therapy. Thus, the role of these therapies in the risk of SM cannot be elucidated and is a limitation of the current analyses. Data pertaining to HR status within SEER dates back only to 1990, limiting the number of women with complete HR status. In addition, comorbidities and additional risk factors known to influence cancer incidence, such as body mass index, tobacco or alcohol consumption, physical activity, use of exogenous hormones, diet, and medications or supplements were not controlled-for in this analysis because the SEER database does not capture this information. Other limitations of the SEER (Curtis 2006) that might lead to overestimation or underestimation of the risk of SM include medical surveillance bias (greater surveillance of women with breast cancer compared to the general population leading to ascertainment bias), multiple comparisons leading to statistically significant increased risk that may have occurred by chance alone, and the contamination of the risk of SM by distant recurrence which can be falsely coded as second cancer, as well as underreporting of treatment received such as radiation, and lack of capture of SM in index cases who leave the original SEER geographic location may also have confounded our results.

Testing for genetic predisposition to inherited cancer syndromes is evolving rapidly (Tung et al. 2015); however, black women are under-represented in studies evaluating inherited cancer syndromes. In the study by Tung et al. (2015), black women represented only approximately 5 % of the individuals tested. Our finding that black women are at an increased risk of second cancer suggests that this population would likely benefit from more genetic evaluation and counseling after a primary breast cancer diagnosis. Race is currently not a factor in NCCN guidelines for genetic counseling, but based on our findings, consideration should be given towards including race in evaluating genetic breast cancer risk.

Furthermore, with the increasing use of germline panel testing for inherited cancer syndromes, there is a clinical need for correlation between genotype and phenotype. We recommend that large databases such as SEER consider collecting data on BRCA1 and BRCA2 and other inherited cancer predisposition genes, since the large clinical information available in SEER provides an excellent opportunity for a better understanding of the risk of SM based on specific DNA repair genes.

The search for biological surrogate markers to identify tumors with abnormalities in DNA repair is underway (Watkins et al. 2014). Homologues recombination deficiency (HRD) score is a potential biological maker for impaired DNA repair (Watkins et al. 2014). A potential correlation between the HRD score of a primary breast cancer and risk of secondary cancers deserves future exploration. The ability to identify patients who might be at higher risk for SM can have implications for primary therapy for the initial breast cancer, such as consideration for prophylactic surgery.

## References

[CR1] Bartkova J, Tommiska J, Oplustilova L, Aaltonen K, Tamminen A, Heikkinen T, Mistrik M, Aittomaki K, Blomqvist C, Heikkila P, Lukas J, Nevanlinna H, Bartek J (2008). Aberrations of the MRE11-RAD50-NBS1 DNA damage sensor complex in human breast cancer: mRE11 as a candidate familial cancer-predisposing gene. Mol Oncol.

[CR2] Berrington de Gonzalez A, Curtis RE, Gilbert E, Berg CD, Smith SA, Stovall M, Ron E (2010). Second solid cancers after radiotherapy for breast cancer in SEER cancer registries. Br J Cancer.

[CR3] Bhaskaran K, Douglas I, Forbes H, dos-Santos-Silva I, Leon DA, Smeeth L (2014). Body-mass index and risk of 22 specific cancers: a population-based cohort study of 5.24 million UK adults. Lancet.

[CR4] Clarke CA, West DW, Edwards BK, Figgs LW, Kerner J, Schwartz AG (2003). Existing data on breast cancer in African-American women: what we know and what we need to know. Cancer.

[CR5] Curtis RE (2006). New malignancies among cancer survivors : SEER cancer registries, 1973–2000.

[CR6] Daly B, Olopade OI (2015). A perfect storm: how tumor biology, genomics, and health care delivery patterns collide to create a racial survival disparity in breast cancer and proposed interventions for change. CA Cancer J Clin.

[CR7] Iqbal J, Ginsburg O, Rochon PA, Sun P, Narod SA (2015). Differences in breast cancer stage at diagnosis and cancer-specific survival by race and ethnicity in the United States. JAMA.

[CR8] John EM, Miron A, Gong G, Phipps AI, Felberg A, Li FP, West DW, Whittemore AS (2007). Prevalence of pathogenic BRCA1 mutation carriers in 5 US racial/ethnic groups. JAMA.

[CR9] Kurian AW, Fish K, Shema SJ, Clarke CA (2010). Lifetime risks of specific breast cancer subtypes among women in four racial/ethnic groups. Breast Cancer Res.

[CR10] Mellemkjaer L, Friis S, Olsen JH, Scelo G, Hemminki K, Tracey E, Andersen A, Brewster DH, Pukkala E, McBride ML, Kliewer EV, Tonita JM, Kee-Seng C, Pompe-Kirn V, Martos C, Jonasson JG, Boffetta P, Brennan P (2006). Risk of second cancer among women with breast cancer. Int J Cancer.

[CR11] Molina-Montes E, Requena M, Sanchez-Cantalejo E, Fernandez MF, Arroyo-Morales M, Espin J, Arrebola JP, Sanchez MJ (2015). Risk of second cancers cancer after a first primary breast cancer: a systematic review and meta-analysis. Gynecol Oncol.

[CR12] Nanda R, Schumm LP, Cummings S, Fackenthal JD, Sveen L, Ademuyiwa F, Cobleigh M, Esserman L, Lindor NM, Neuhausen SL, Olopade OI (2005). Genetic testing in an ethnically diverse cohort of high-risk women: a comparative analysis of BRCA1 and BRCA2 mutations in American families of European and African ancestry. JAMA.

[CR13] Renehan AG, Tyson M, Egger M, Heller RF, Zwahlen M (2008). Body-mass index and incidence of cancer: a systematic review and meta-analysis of prospective observational studies. Lancet.

[CR14] Schaapveld M, Visser O, Louwman MJ, de Vries EG, Willemse PH, Otter R, van der Graaf WT, Coebergh JW, van Leeuwen FE (2008). Risk of new primary nonbreast cancers after breast cancer treatment: a Dutch population-based study. J Clin Oncol.

[CR15] Tammemagi CM, Nerenz D, Neslund-Dudas C, Feldkamp C, Nathanson D (2005). Comorbidity and survival disparities among black and white patients with breast cancer. JAMA.

[CR16] Tung N, Battelli C, Allen B, Kaldate R, Bhatnagar S, Bowles K, Timms K, Garber JE, Herold C, Ellisen L, Krejdovsky J, DeLeonardis K, Sedgwick K, Soltis K, Roa B, Wenstrup RJ, Hartman AR (2015). Frequency of mutations in individuals with breast cancer referred for BRCA1 and BRCA2 testing using next-generation sequencing with a 25-gene panel. Cancer.

[CR17] Watkins JA, Irshad S, Grigoriadis A, Tutt AN (2014). Genomic scars as biomarkers of homologous recombination deficiency and drug response in breast and ovarian cancers. Breast Cancer Res.

